# Health System Responses to the Health Needs of Refugees and Asylum-seekers in Malaysia: A Qualitative Study

**DOI:** 10.3390/ijerph16091584

**Published:** 2019-05-06

**Authors:** Fiona Leh Hoon Chuah, Sok Teng Tan, Jason Yeo, Helena Legido-Quigley

**Affiliations:** 1 Saw Swee Hock School of Public Health, National University of Singapore, 12 Science Drive 2 #10-01, Tahir Foundation Building, Singapore 117549, Singapore; fionachuah@u.nus.edu (F.L.H.C.); soktengtan@u.nus.edu (S.T.T.); 2 United Nations High Commissioner for Refugees Malaysia, 570, Jalan Bukit Petaling, Bukit Petaling, Wilayah Persekutuan Kuala Lumpur, Kuala Lumpur 50460, Malaysia; YEOJ@unhcr.org; 3 Department of Nutrition and Dietetics, Universiti Putra Malaysia, Jalan Upm, Serdang 43400, Selangor, Malaysia; 4 Department of Global Health and Development, London School of Hygiene and Tropical Medicine, London WC1H 9SH, UK

**Keywords:** Malaysia, refugees, asylum-seekers, urban refugees, health needs, health systems, health policy, forced migration

## Abstract

*Background*: This study was conducted to examine the responses and challenges in addressing the health needs of refugees and asylum-seekers in Malaysia from a health systems and policy perspective. *Methods*: Twenty semi-structured in-depth interviews were conducted with key informants comprising experts, healthcare professionals and program personnel with professional experience in refugee health issues. Deductive and inductive analyses were conducted to identify themes. *Results*: Our study identified a broad range of actors involved in the response to refugee health locally, of which a greater alignment of interests, collaboration and sharing of responsibility is needed. From a health systems and policy perspective, financial constraints are among the key challenges in addressing the health needs of the refugee and asylum-seeker population in Malaysia. While participants reported high quality healthcare being present in Malaysia, this was not affordable to refugees and asylum seekers. Cultural and language discordance are also key challenges faced by healthcare workers in the delivery of services; accentuating the need for greater cultural competence and language support. Improved access to medication is needed for those with chronic illnesses in order to effectively address the comprehensive health needs of the refugee and asylum-seeker population. *Conclusions*: Suggested ways forward include adopting a comprehensive health advocacy strategy grounded in the right to healthcare for all; adopting a multi-sectoral approach; tackling the social determinants of health; seeking diversified funding at the global and national level; and improving coordination and collaboration between the various actors.

## 1. Background

Malaysia is an upper-middle-income country situated in Southeast Asia [1]. Due to its’ geographic location and economic development, Malaysia has emerged as a destination for many groups of migrants [2]. Refugees and asylum-seekers fleeing persecution, war and violence have also escaped to Malaysia throughout the past decades. Since the mid-70s, Malaysia has hosted large numbers of refugees and asylum-seekers, including thousands of Vietnamese who were granted temporary refuge in camps while they were processed for resettlement by the United Nations High Commissioner for Refugees (UNHCR) [3]. Throughout the 1980s and 1990s, Malaysia also received over 50,000 Filipino refugees, several thousand Muslim Chams from Cambodia, and a few hundred Bosnian refugees who were allowed to settle in the country [3]. At present, there are some 164,620 refugees and asylum-seekers registered with UNHCR in Malaysia, of whom 86% originate from Myanmar and the remaining from countries including Syria, Iraq, Somalia, Yemen, Afghanistan, Palestine, Iran and Sri Lanka [4].

Despite its history of hosting refugees, Malaysia is not a state party to the 1951 Convention Relating to the Status of Refugees and its’ 1967 Protocol [5,6]. It also lacks the legislative and administrative frameworks to effectively manage refugees and asylum-seekers in the country [3]. By law, refugees and asylum-seekers are not differentiated from undocumented migrants who make up a third of the migrant population [3]. However, refugees recognised by UNHCR Malaysia possess a “de facto” status which allows them to remain in the country, but without official protection from any national legislation [7]. This situation of mixed-migration adds much complexity to refugee protection [8] and in the absence of legal frameworks, the population has no formal access to basic education and work rights [9]. Unlike previous instances where refugees and asylum-seekers were placed in camps in Malaysia, the population now lives in urban settings amongst host communities and other migrant groups [10]. While this has its advantages, urban living brings about a different set of protection, social and health challenges among the refugee and asylum-seeker population [11].

In addition to the health risks associated with the perilous journeys of fleeing violence and persecution [12], refugees and asylum-seekers are often faced with epidemics and acute malnutrition during the initial phase of a crisis [13]. An epidemiological transition occurs when a crisis is protracted resulting in a double burden of disease with increased prevalence of non-communicable diseases such as hypertension, diabetes, musculoskeletal diseases, chronic respiratory diseases and mental health conditions [14,15]. This is further complicated by issues of population ageing, urbanization and the increase in asylum-seekers fleeing with chronic conditions requiring immediate and long-term care and management upon arrival [14,16]. Similarly, refugees and asylum-seekers in Malaysia are subject to such conditions and many develop chronic diseases throughout their protracted periods of stay [16]. A comprehensive health response with necessary adaptations is thus necessary to meet the existing health demands of the refugee and asylum-seeker population.

The Malaysian health system consists mainly of a tax-funded public sector and a private sector that generates revenue through fee-for-service, out-of-pocket payments, and private insurance [17]. Malaysia is widely known for its successes in improving population health at low costs through universal and comprehensive service provision [17]. However, the public healthcare sector limits its low-cost services to citizens only and imposes high fees upon foreigners who seek medical care [18]. In advocating better access to healthcare for refugees, UNHCR Malaysia successfully negotiated a formal Memorandum of Understanding with the Ministry of Health (MoH) in 2005, which stipulates that all refugees recognised by UNHCR would be given a 50% discount off the foreigners’ rate at public healthcare facilities [10,19,20]. Despite this discount, the cost of medical care is still exorbitant resulting in significant financial barriers in accessing basic healthcare services [16]. The high healthcare cost was further exacerbated following a 2014 amendment to the 1951 Medical Fees Act involving a 100% increase in medical charges for all foreigners [20]. Additionally, healthcare access among refugees and asylum-seekers is often compounded by other barriers in the form of security and protection problems, language difficulties, and poor health literacy [21].

Due to the complex social and policy environment for refugees and asylum-seekers in Malaysia, little is known on the capacity of the health system and the challenges that local and international actors face in responding to the health needs of the population. Further, the paucity of academic research examining forced migration and health issues in Malaysia can compromise efforts in identifying potential solutions to the policy and practical challenges of responding to these needs. To address this gap, this qualitative study was conducted to examine the health systems responses and challenges in addressing the health needs of refugees and asylum-seekers in Malaysia from the perspectives of healthcare practitioners, program personnel and experts on refugee health.

## 2. Methods

This study was conducted from July 2016 to January 2018 as one of the outputs of a research project exploring the health issues of refugee and asylum-seeker populations and its responses in Southeast Asia. This paper focuses on Malaysia in particular, examining the health systems’ responses to the health needs of refugees and asylum-seekers residing in the country and the challenges that local and international actors face in responding to the health needs of the population. In a companion qualitative paper, the key health concerns and access barriers among the refugee and asylum-seeker population in Malaysia are examined and reported [22].

### 2.1. Participants and Sampling

The researchers conducted purposive sampling to identify key informants for recruitment. Participants included experts, healthcare workers and program personnel with professional expertise and experience working on refugee health issues in Malaysia. They were from UN agencies, public healthcare facilities, international and civil society organizations and academic institutes. Additional participants were further recruited through snowball sampling techniques based on nominations by the initial batch of participants. The researchers stopped recruitment when it was collectively agreed that thematic saturation was achieved. In total, 20 participants were recruited and interviewed, including 12 who were based in Malaysia and the others from countries in the region who had relevant professional expertise. The sample characteristics are shown in Table 1.

### 2.2. Ethics

Ethical approval for the research project of this study was obtained from the National University of Singapore Institutional Review Board (NUS-IRB) (NUS IRB Reference Code: B-16-126). When recruiting, all potential participants were given an information sheet regarding the study. The researchers obtained signed consent from all recruited participants for their agreement on the participation in the research project, the audio-recording of interviews, and the possibility of being quoted anonymously in research outputs. They were provided the option to refuse any questions posed to them or withdraw from the project at any point in the research. All identifying data was removed from the audio-recording and transcripts to ensure anonymity of participants and confidentiality of the information provided. Due to the sensitivity of the topic, the researchers sought professional feedback and advice from participants and other experts in the field on the best ways of presenting the study findings. This was done to minimize any potential harm that the study findings could bring upon the situation of refugees and asylum-seekers in Malaysia.

### 2.3. Data Collection

In-depth interviews were conducted face-to-face or via Skype with participants at a time and site of the participants’ preference. Each interview lasted approximately 60 minutes. A semi-structured topic guide was used to explore the health systems responses to the health issues of refugees and asylum-seekers in Malaysia. The line of questioning was adapted according to the participants’ responses and prompts were introduced when necessary to ensure all topic areas were covered to meet the study aim. All audio recordings were transcribed in verbatim. One participant chose not to be audio-recorded, hence field notes were taken and typed into a note sheet following the interview.

### 2.4. Data Analysis

An interpretive approach was adopted in the coding and analysis of the transcripts and field notes. This approach focuses on how participants interpret, perceive and make sense of their experiences [23]. Thematic analysis was done to identify key themes with the World Health Organization (WHO)’s Building Blocks of Health Systems Framework [24] in mind. Inductive analysis was done simultaneously to identify emergent themes apart from those that were established based on the framework. QSR NVIVO11 was used throughout the analysis process to store and manage the data. The researchers developed and reviewed the themes through an iterative process involving regular discussions to build consensus on the final list of themes and its findings. Deviant cases were sought out, which included individuals presenting ideas or topics that were distinct from the majority. To improve credibility, participants were approached for a member check at the final stage of preparing the manuscript to validate the findings for an accurate representation of their perspectives.

### 2.5. Definition and Conceptual Framework

Based on WHO’s definition, ‘health system’ is defined in this paper as (1) ‘all activities whose primary purpose is to promote, restore and/or maintain health’ and (2) ‘the people, institutions and resources arranged together to improve the health and wellbeing of the population they serve, while responding to the people’s expectations and protecting them against the cost of ill-health’ [25]. In Malaysia, the population served by the health system includes refugees and asylum-seekers, while the actors responding to their health needs encompass stakeholders from the public, private and civil society sector. In line with this definition, the WHO Building Blocks of Health Systems Framework was used to guide the analysis in examining the health systems’ response to the health needs of refugees and asylum-seekers. This framework described health systems as comprising of six key components: (1) service delivery; (2) health workforce; (3) information and research; (4) medical products and technologies; (5) healthcare financing; (6) governance and leadership [24]. In addition, inductive analysis was also conducted to identify emergent themes.

## 3. Results

The findings of this study are based on the perspectives of our participants and will be presented under three sections. Section 3.1 describes the key actors who comprise the health system in responding to the health needs of refugees and asylum-seekers in Malaysia. Section 3.2 presents the health systems responses to the health needs of refugees and asylum-seekers in Malaysia, as analysed in accordance with the WHO Building Blocks of Health Systems Framework. Section 3.3 reports on the potential strategies and ways forward in addressing refugee health issues in Malaysia.

### 3.1. Key Actors 

Participants identified a number of key health system actors in the overall response towards refugee health issues in Malaysia. These actors can be broadly categorized as: (1) the state government which constitutes the MoH, all public healthcare facilities and other relevant ministries which have an indirect influence on refugee health issues; (2) the civil society which consists mainly of international and local non-governmental organizations (NGOs); (3) the UNHCR; (4) the refugee communities; (5) the private sector which comprises private health facilities and private entities supporting the cause; and (6) academia which includes academic and research institutes.

#### 3.1.1. Roles and Involvement of Key Actors

All participants considered the state government as the main decision-maker for national level refugee-related policies. In the context of healthcare delivery, most participants described MoH as the largest service provider and the main health partner for UNHCR. Generally, a large majority of refugees and asylum-seekers sought healthcare at public health facilities particularly when they required acute care and/or secondary and tertiary care. As reported during the study period, the Ministry of Finance and the Immigration Department of the Ministry of Home Affairs were considered key actors given their indirect influence on refugee health issues. The Ministry of Finance determined the budget for the MoH, and thus had some effect on matters of health financing concerning the migrant population including refugees and asylum-seekers. The immigration department of the Ministry of Home Affairs was said to govern all issues relating to the legal status of refugees and asylum-seekers in Malaysia, which is a key determinant of health for the population. In recent years, a Malaysia-UNHCR Joint Task Force comprising six government ministries and UNHCR was established to address refugee issues at the national level.

According to most participants, civil society was perceived to play a major role in filling the service delivery gaps in primary healthcare by offering affordable healthcare services that are tailored to the refugee and asylum-seeker population. Apart from clinical services, a few NGOs had run health promotion activities comprising the dissemination of health information including how to navigate the health system; as well as health education and prevention programs for refugee communities. Additionally, a few NGOs were cited as active advocates for the rights of refugees and asylum-seekers. Some NGOs provided shelter, financial assistance and social support to the population. The health NGOs also commonly conducted case referrals to public health facilities.

The UNHCR was described by most participants as the main intergovernmental organization mandated to provide international protection to refugees in Malaysia. Their key areas of work ranged from providing assistance and protection, to finding durable solutions for refugees. In the context of health, UNHCR was cited as the lead agency in coordinating all health stakeholders involved in providing health-related services to the refugee population. This involved active engagement with the MoH, civil society, refugee communities, the private sector and academia for purposes of service delivery, case referrals, health financing and advocacy. According to many participants, the collaboration between the various agencies in the health sector had improved over the years due to UNHCR’s efforts in bringing stakeholders together through various platforms. In addition, UNHCR was also cited as a funder of several health projects implemented by other actors.

The refugee communities were described to have a major role in supporting refugees and asylum-seekers in accessing healthcare. Such support was usually provided through community leaders, community health workers and/or medical coordinators from refugee community-based organizations (CBOs), NGOs or UNHCR. Apart from providing health education to the communities, they also facilitated healthcare seeking processes, for instance through the provision of translation services or by acting as liaisons for refugees or asylum-seekers seeking healthcare at hospitals. In addition, refugees and asylum-seekers who needed medical treatment often turn to their community members and CBOs for financial support. Some of the more established CBOs also provided financial assistance to its members for medical treatment through crowdfunding mechanisms.

As pointed out by several participants, private health facilities were also an avenue for refugees and asylum-seekers to seek healthcare. Over the years, a number of private hospitals have extended services such as cardiac operations to refugee patients who were not able to access such treatment at public health facilities. A few participants also highlighted a partnership that UNHCR had formed with a chain of private clinics to offer primary healthcare at affordable rates to refugees. It was also reported that a financial institute in Malaysia had partnered with UNHCR to offer healthcare insurance to refugees. Private companies have also donated funds for refugee-related projects and provided ad-hoc support in the form of medical and nutritional products as part of their corporate social responsibility (CSR).

**Figure 1:**
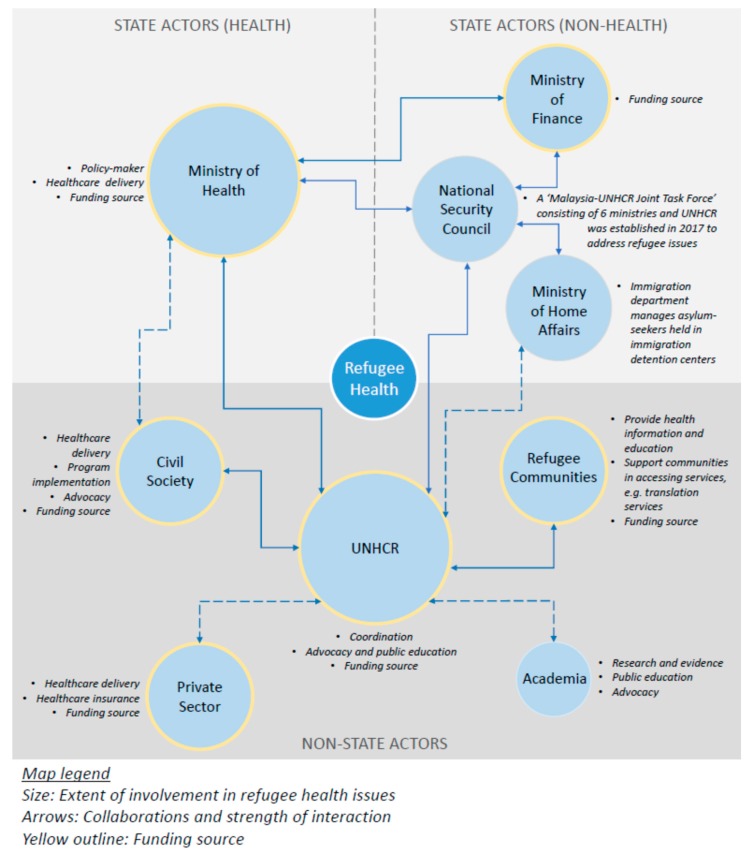
Stakeholder mapping of refugee health actors in Malaysia.

Some participants indicated that there has been increasing interest among the academic sector on refugee issues both in Malaysia and in the region. Primarily, the academic sector’s involvement includes conducting research and generating evidence on refugee related topics. A small number of academicians have also been involved in advocacy efforts. As highlighted by a few participants, academic actors play an important role in educating the public as well as the younger generation about refugee-related issues in Malaysia. Figure 1 provides a snapshot of the various actors, their collaborations and the main roles they had in relation to refugee health as reported by participants during the study period.

#### 3.1.2. Challenges Faced by Key Actors

Given the complex health needs of the refugee and asylum-seeker population, and the difficult environment in which health system actors operate, participants’ narratives highlight multiple challenges encountered by key actors in responding to health issues of the population. Table 2 presents these challenges by sector as described by the participants.

### 3.2. Health Systems Responses

The WHO Building Blocks of Health Systems Framework [24] underpinned the overall analysis of the health systems responses to refugee health issues in Malaysia. The six key components of the health systems framework and the description of related findings are presented in Table 3.

#### 3.2.1. Service Delivery

• Good availability and quality of services

According to most participants, public health facilities and services in Malaysia were considered widely available, particularly in urban settings where a large majority of refugees and asylum-seekers reside. Some participants also added that the Malaysian health system is regarded highly internationally. Participants also expressed that refugees have been given reasonable healthcare treatment despite the overall legal circumstances for refugees and asylum-seekers in Malaysia. Nonetheless, limited accessibility to healthcare services remain a problem among refugees and asylum-seekers due to the many barriers encountered.

*“All in all, the capacity is there and then the quality and quantity of clinics are also there. So, I don’t see an issue about supply and demand but I see an issue probably more of accessibility due to the high cost […] Availability is there but the affordability is not there.” *

*(I01)*

Many participants also spoke about NGO health clinics, that despite its limited availability, these clinics were easy to reach as most facilities are located in vicinities close to refugee communities. In such settings, the protection and financial barriers of accessing healthcare services are mitigated, as documents are not required at point of care and cost of treatment is free or minimal. 

• Service provision beyond basic essential healthcare services

Beyond the availability of basic healthcare services including acute care and primary healthcare, the provision of comprehensive health needs for the refugee population was perceived as inadequately addressed. As described by some participants, a significant proportion of the refugee and asylum-seeker population reside in Malaysia for protracted periods while awaiting processing by UNHCR or due to a lack of durable solutions. According to participants, addressing the comprehensive health needs of refugees entail ensuring that the population has access to various services throughout the continuum of care including mental health and psychosocial support; antenatal care; preventive care and health education; family planning; as well as rehabilitative and palliative care.

*“[…] comprehensive health needs are still existent, being that the population is going to be here for a protracted period of time, we can’t just focus on access to just basic services; we also need to look at the more comprehensive needs for the long-term, which will then include all the other areas”*

*(I09)*

• Cultural and language differences as barriers to service delivery 

Despite the availability of healthcare services in Malaysia, some participants pointed out barriers relating to the cultural backgrounds and practices of refugee and asylum-seeker patients. It was reported that patients in some instances have encountered prejudices due to differences in culture and nationality. As described by participants, language barriers precipitates communication challenges between healthcare providers and patients, particularly new arrivals who are foreign to local languages. This was cited as a problem in various processes including medical history taking, acquiring consent for treatment, developing treatment plans, and discussing financial or administrative issues.

*“When we talk about refugee health in Malaysia, we’ll find a certain phenomenon. There’s of course the cultural issue, there’s the language issue, and there’s discriminatory issues.”*

*(I19)*

#### 3.2.2. Health Workforce

• Human resource challenges in the context of treating refugees

In terms of human resource capacity, responses were mixed. Some participants mentioned that manpower is lacking in some public healthcare facilities and described healthcare workers as overworked due to high patient loads. Other participants however, argued that oversupply of doctors and nurses, was evident in Malaysia. A few participants iterated that human resources may be limited in public healthcare facilities situated in central locations like the Klang Valley where refugee and asylum-seeker populations are concentrated. Some participants also shared that specialist centres for infectious diseases, particularly those treating a high caseload of refugee patients with TB and HIV, were understaffed. The lack of human resource was considered a primary factor limiting the ability of healthcare providers to expand programs and services.

*“I think basically because Klang Valley has such a concentration of refugees here, I would think that some of the larger hospitals are struggling a little bit, in terms of manpower and meeting the needs of refugees.”*

*(I09)*

• The influence of professional norms

Most participants felt that a good number of healthcare workers including doctors, nurses and social workers in the public sector and civil society sector remain altruistic and committed to providing quality healthcare to refugees and asylum-seekers in accordance with their duty to care. Many of them maintain a medical and humanitarian stance in treating their patients irrespective of legal status and ability to pay. In one example provided, local staff nurses in a public healthcare facility even took the initiative to learn words and sentences in the language of refugee patients to communicate better with them.

*“[Refugees] don’t have proper documentation […] from the medical point of view, health staff are supposed to provide medical treatment without taking into consideration criminality or legality.” *

*(I01)*

Some participants as healthcare workers themselves, emphasized that they have a responsibility to be a voice for the vulnerable, disadvantaged and marginalized. However, their ability to treat was at times undermined by immigration laws dictating the management of patients without identity documents. Nonetheless, most participants iterated that the decision to treat or admit a patient should be a clinical decision rather than an administrative one. In addition, some participants perceived that providing treatment to refugees conforms with the greater good of protecting national public health interests.

*“[…] as a health professional, we’re also the voice for people who are marginalized, in terms of access to healthcare. If we don’t speak up for them, then who does, you know? And if we don’t put the stance and fight for those who are without a voice, then who else will do that? Because we are the ones who know what the needs are of a person who’s sick and vulnerable.” *

*(I09)*

#### 3.2.3. Information and Research

• Unavailability of health information and limited funding for research

Some participants highlighted that there has been limited availability of public data or information on the health status of refugees and asylum-seekers in Malaysia. Participants also pointed out the lack of information sharing of health-related data between the various actors. In the context of research, some participants stated that despite increasing interest among academics and research institutes on the refugee issues, research in this area remain a challenge due to limited funding and availability of data.

*“[Funders] will never give you money for [refugee] work. They won’t. That’s what stops the lecturers from getting or working with UNHCR […] Oh, you’ll never get the funding for it.” *

*(I04)*

#### 3.2.4. Medical Products and Technologies

• Limited access to medication leading to poor adherence

Some participants mentioned that following the policy changes in recent years, foreign patients were only prescribed limited days of medication when seeking treatment at public healthcare facilities. Many participants raised concerns that this would jeopardize treatment adherence among refugee and asylum-seeker patients particularly those with chronic conditions as they may not be able to afford more medication from private pharmacies. As emphasized by a number of participants, the lack of access to medication can be detrimental to secondary prevention of diseases among refugee and asylum-seeker patients, leading to poorer health outcomes and costlier treatment in the long run.

*“So, antibiotics, chronic diseases, everything will have only five days of supply. Meaning that, if they have diabetes, heart problems […] you have to pay for the five days, at the same time, you have to find your supply from pharmacy outside for the additional medication. Basically, the long-term care, quality, everything would be diminished, especially for those financially challenged; and poor control of diabetes which may lead to a lot of long-term complications.”*

*(I01)*

#### 3.2.5. Healthcare Financing

• Healthcare financing as a major challenge in responding to refugee health issues

Many participants perceived that the greatest challenge of providing refugee-inclusive healthcare in Malaysia linked to issues of financing. Due to national budgetary constraints, healthcare in Malaysia was perceived as a commodity that should be prioritized for local citizens. As such, healthcare costs for foreigners, particularly at the secondary and tertiary level, remain largely unaffordable to the refugee and asylum-seeker population. Most participants iterated that despite the 50% discount accorded to refugee patients at public healthcare facilities, the out-of-pocket expenditure for them is still exorbitant and unaffordable. The cost is greater for those without a UNHCR card.

*“The government is also facing difficulties in trying to manage with the restricted budget they have […] I think what they did is probably something that would need to be done for them in managing and ensuring that healthcare is prioritized for Malaysian citizens. If they can’t even have enough for the Malaysians, how are they going to deal with the rest?”*

*(I09)*

Healthcare insurance for refugees were perceived by many participants as a promising initiative to reduce barriers of affordability in accessing inpatient care. However, the implementation of the insurance scheme—REMEDI, which was initiated by UNHCR in collaboration with a private company, had been met with various challenges including the slow uptake among the population. Additionally, some participants also stated that the medical coverage for REMEDI was insufficient as it did not cover outpatient treatment.

*“It’s a good emergency sort of insurance policy, but the uptake is not that good […] the coverage is not that high because, some of them, first of all, they may not be aware.”*

*(I01)*

#### 3.2.6. Leadership and Governance

• Lack of overall governance and policy on refugee health

Many participants pointed out the absence of governance structures and targeted policies for refugee health in Malaysia. Consequently, the processes of treating refugee and asylum-seeker patients have not been standardized across public healthcare facilities. A few participants also commented on the overall lack of common understanding and communication between policy-makers and healthcare personnel directly involved in the care and management of refugee and asylum-seeker patients.

*“[…] most policy is not inclusive of refugees’ health. That’s why we don’t have, like, written policy for pregnant women with HIV, you know what I mean? They don’t spell it out, so some hospitals decide, ’Never mind, they are refugee […] they are foreigners, so cannot get free.’ ”*

*(I10)*

• Impact of the broader policy environment on refugee health

Many participants commented on the policy and protection environment which has a significant impact on the health and wellbeing of refugees and asylum-seekers. As described by participants, the refugee issue is commonly treated as an ‘illegal immigrant’ issue. Their lack of legal status which denies them the right to work and access to education serves as determinants that are detrimental to their health and wellbeing living in Malaysia. A few participants also emphasized that the constant fear of arrest and the inability to support oneself also leads to significant impact on the psychological wellbeing of refugees and asylum-seekers.

*“I think in terms of social determination of health, […] if we look at the intervention pyramid of mental health and psychosocial support, the bottom is always actually the basic need, which for me is a social determination of health. So if let’s say all the refugees already can have a sufficient achievement of their basic need, that already covers most of the social determinants, and health needs itself. For example, like education, employment, security.”*

*(I07)*

### 3.3. Potential Strategies and Ways Forward

The participants’ narratives presented some overarching ideas of potential strategies and ways forward in addressing refugee health issues in Malaysia. These emerged as four key themes that highlight the participants’ perspectives on approaches that can be used to inform policy and interventions aimed at improving the overall response to refugee health issues in Malaysia. 

#### 3.3.1. A Comprehensive Health Advocacy Strategy

Many participants highlighted the need for a comprehensive advocacy strategy to address key health issues relating to the refugee and asylum-seeker population in Malaysia. One of the key suggestions was to implement an organized *bona fide* body of council consisting of relevant stakeholders to champion the rights and wellbeing of refugees and asylum-seekers in Malaysia. The participants drew from the best practices of governance and coordination for HIV in Malaysia, citing the establishment of Malaysian AIDS Council which is perceived to have the biggest influence in regard to issues relating to HIV and AIDS in the country.

*“[...] maybe if the NGOs, UNHCR can organize very much in the same line as Malaysian AIDS Council, a refugee council of NGOs, of government entities, maybe they will have more say […] I think that might be the way to go because Malaysian AIDS Council has the biggest say when it comes to AIDS.”)*

*(I04)*

In advocating for the health rights of refugees and asylum-seekers in Malaysia, many participants also emphasized the need to include such vulnerable populations in the overall provision of Universal Health Coverage (UHC) at the country level. Participants asserted that UHC could be a potential approach in advocating for equitable healthcare among the refugee and asylum-seeker population on the basis that access to healthcare is a fundamental human right. This could incorporate the introduction of alternative health financing mechanisms including different models of healthcare insurance for refugees.

*“I think […] access to health is part of the human right, and a refugee is a human being. I think this is a basic fundamental concept that we have to understand. So it’s not to say that when they are not considered as a citizen of the country, then they shouldn’t have any basic right […] access should be whenever the health services are available that it should be able to be accessed by refugees.”*

*(I07)*

Within the context of proposing a comprehensive advocacy strategy for refugee health, many participants spoke about the need for targeted efforts in public sensitization of refugee-related issues for greater awareness through forums, dialogues, campaigns and media platforms. Participants explained that the level of public awareness needs to increase in order for advocacy efforts to be mobilized further. Other participants proposed to engage interested parties from civil society or academic sectors to lead on advocacy efforts. One participant highlighted the potential usefulness of evidence from research to support such advocacy work. Overall, it was further emphasized that there is a need to change negative public perceptions on refugees and asylum-seekers by increasing awareness on the plight of the population as well as their social and economic contributions to the country.

*“They are working very hard for the economy of the host country by doing informal jobs, informal employment, especially those jobs not taken up by the locals – like, the dirty, difficult, dangerous jobs […] and they don’t have a proper insurance to cover as well. They actually sacrifice a lot for the country without taking into consideration what the host country is doing to them. This is the tolerance that they do because they are allowed to be here while supporting the growth and development of the country.”*

*(I01)*

Some participants also suggested more training and sensitization efforts for public healthcare providers. One participant recommended greater advocacy with state actors to promote inclusivity of refugee and asylum-seeker populations in national public health policies and health information systems, as these would yield long-term benefits for population health. Additionally, a refugee-friendly environment in public healthcare facilities was advocated for by many of the participants. Existing best practices include having signboards and posters in languages of the refugee communities at public healthcare facilities with large refugee patient load, and interpreter support. Some participants also suggested that these practices could be accompanied with guidelines or codes of conduct for refugee patient management and referrals. 

#### 3.3.2. Tackling the Social Determinants of Health

As iterated by most participants, addressing the social determinants of health is fundamental to improving healthcare access and ultimately, health outcomes among the refugee and asylum-seeker population in Malaysia. As stated above, financial difficulties were presented as a key barrier in acquiring healthcare services. Participants reasoned that granting refugees and asylum-seekers the basic right to employment by integrating them into the formal workforce will improve their earning power thus enabling them to afford healthcare services. Apart from improving healthcare access among the population, this could also potentially reduce the amount of healthcare debt derived from services delivered to the population. As iterated by one participant, refugees and asylum-seekers can assume greater responsibility in making better decisions for their health with greater financial capital.

*“So, the solution would not solely come from the health sector [...] it’s also the social determinants. They have to have access to livelihood - formal livelihood that would allow them that choice [...] to make that decision about what they spend money on.” *

*(I09)*

In tackling the social determinants of health, most participants spoke about the importance of granting refugee children access to formal education. Not only would this improve the level of health literacy among the young but also, many participants perceived education as key to resolving poverty among the population in the long run, thus improving their economic status and ability to afford healthcare. One participant stressed that educating the young includes imparting soft-skills such as coping strategies, problem-solving and critical thinking, which enhances their emotional intelligence and boosts one’s mental wellbeing. Overall, most participants perceived that tackling the social determinants of health, particularly in regard to ensuring formal employment and education, would overcome numerous barriers to healthcare access and improve the physical and psychological wellbeing of the population as a whole.

#### 3.3.3. Diversified Funding for Better Health Financing

In regard to solutions relating to the health system, most of the participants’ responses were associated with ideas to improve healthcare financing for refugees. A main viewpoint was related to diversifying the funding sources for refugee health issues by involving both the civil sector and private sector. Existing successes include cost-sharing models of funding between NGOs and UNHCR, as well as engagements with the private sector through public private partnerships to raise and channel funds as part of their CSR.

*“It can be within the private hospital itself, or it can be with the company where they generate income […] Then you can have the financing power from the income-generating entity to put into healthcare. We can also see there are many examples in public and private partnerships. There are some programs, for example, like medication with the private pharmaceutical company […] or it can be some other services provided with the CSR policy.”*

*(I07)*

Many participants spoke about REMEDI, the health insurance program for refugees that was initiated through partnerships between UNHCR and private companies. This program was considered by many participants as a timely initiative due to surges in medical costs for foreigners receiving treatment at public hospitals. Participants considered this a promising effort in extending social protection in health to the refugee and asylum-seeker population, although challenges still remain in relation to its limited medical coverage and uptake.

#### 3.3.4. Improved Interagency Coordination and Collaboration

Most participants suggested that greater coordination; facilitated through effective and efficient communication between all state and non-state actors could improve the overall response to refugee health issues in Malaysia. This includes the formation of new and innovative partnerships between various sectors including the private sector. According to participants, examples that have emerged in recent years include partnerships between UNHCR and private entities to offer healthcare insurance for refugees; affordable primary and secondary care; and medications procured at a cheaper rate.

*“[...] most of our projects are being funded by private sector. You come up with a good proposal which will benefit the beneficiaries. I’m sure... because there are a lot of tax incentives by doing such... I mean CSR.” *

*(I14)*

On the whole, UNHCR is perceived as the key organization in coordinating the various stakeholders and bringing them together for better interagency collaboration on refugee health issues in Malaysia. Most participants felt that there is a need for greater dialogue and stronger collaboration with the state actors, including the non-health related ministries due to the various social and economic factors influencing health for refugees and asylum-seekers in Malaysia. Some participants also spoke about the importance of interagency coordination between NGO clinics and UNHCR in regard to registration for asylum-seeker patients who require further medical treatment at hospitals. Effective coordination between the organizations to facilitate the registration and issuance of a UNHCR document was described as an essential component of the overall referral mechanism to ensure access for refugees at public hospitals. 

## 4. Discussion

This qualitative study examines the health systems responses and key challenges in addressing the health needs of refugees and asylum-seekers in Malaysia. The mapping of key actors identified a range of stakeholders across both health and non-health sectors that have varying roles and involvement in refugee health depending on their mandate and priorities. The key challenge for coordination and collaboration is therefore in aligning interests to achieve agreement on objectives that are mutually beneficial. Across all sectors, financial constraints were identified as a main challenge in addressing the comprehensive health needs of refugees and asylum-seekers in Malaysia. Similarly, a systematic review on non-communicable diseases among urban refugees highlighted financial difficulties as a significant challenge in meeting the increasing demands of urban refugee healthcare [14]. These findings underline the need for diversified funding and partnership for better financial sustainability of refugee health initiatives. On the whole, refugee health responses require responsibility-sharing arrangements across actors and a framework whereby the state can contribute in line with its capacities and receive support in accordance with the level of needs [26]. 

Our findings add to existing literature on the complexities and challenges of healthcare service delivery in urban settings [15,27,28]. In many developing countries, access to secondary and tertiary healthcare was prohibitively lacking [14]. Our findings are in line with such reports which highlight administrative and financial barriers as restricting access to healthcare among populations lacking legal documentation [29,30,31]. As identified in our study, healthcare services are unaffordable for refugees and asylum-seekers despite good availability and quality of healthcare facilities. Consequently, the cost of healthcare remains a key barrier to access among the majority of refugees and asylum-seekers living in Malaysia. Such findings are similar to those reported in other urban studies [17,32,33,34]. Apart from financial considerations, our study identified cultural and language differences as significant barriers in the delivery of healthcare services to refugee and asylum-seeker populations. Similarly, several other systematic reviews have highlighted this as a challenge for healthcare providers [15,27,35]. According to literature, cultural and language discordance may compromise the quality of healthcare, and result in delays in healthcare seeking, reduced detection of illness and missed referrals for further treatment [15,36,37]. Our study accentuates the need for culturally-sensitive healthcare services in promoting better health outcomes among the refugee and asylum-seeker population. In addition to the use of interpreters who are trained to support communication between healthcare providers and patients, it is fundamental to ensure that services match the needs of the refugee and asylum-seeker population [35]. Our companion paper, which described the health needs and access barriers among refugees and asylum-seekers in Malaysia, also highlighted poor health literacy; lack of awareness on one’s right to healthcare; language and cultural differences; protection issues; and an inability to afford healthcare as key barriers to accessing health services [22].

In terms of healthcare workforce capacity, our study identified concerns over the limited human resource in public healthcare settings with higher loads of refugee and asylum-seeker patients. Similarly, a systematic review revealed that in-house constraints resulting from heavy workloads and inadequate human resources, was a prominent concern among healthcare providers offering services to refugees [35]. Despite these limitations, our study showed that there are many healthcare professionals in the public, private and civil society sectors who remain committed to their professional duty of care and undertake a humanitarian stance to provide treatment regardless of a patient’s legal status and ability to afford. Some considered themselves to be advocates of vulnerable populations, while others perceive that this conforms with protecting the public health interests of the national population. These findings are in line with existing literature that highlights the strong influence of professional norms on the behaviours and attitudes of healthcare providers in treating migrant populations despite restrictive legal and healthcare policies [35,38,39]. As identified from our study, cultural competency training for healthcare professionals that begins from their medical training is important to foster greater awareness and sensitivity to the plights and needs of vulnerable populations including refugees and asylum-seekers.

Another key finding from our study is on the need to address the comprehensive health needs of refugees in Malaysia given their protracted displacement in the country. This includes meeting the needs of those with chronic illnesses. Literature has highlighted the inadequacy of healthcare delivery for refugees with chronic illnesses in developing settings including the lack of specialized long-term care, availability of medications, health education and preventive health services [12,40,41]. As pointed out in studies in other settings, the lack of access to regular medication is a potential cause for a higher burden of malnutrition, anaemia, and treatable non-communicable diseases [42,43]. In Malaysia, access to affordable medication for refugee patients can be affected by policy changes. This adds to existing research documenting the difficulties that refugee populations face in accessing essential medication—a common phenomenon even in resettlement countries with good healthcare systems [44]. In our study, the limited access to medications was perceived to have significant implications on refugee patients’ treatment adherence and continuity of care, particularly those with chronic illnesses. While NGO clinics have stepped in to fill this gap, the financial burden of providing long-term care is significant on the sector, which already faces resource limitations.

Our study participants identified a lack of governance over refugee health issues within the policy realm. As evidenced from other settings, implementing refugee-inclusive health policies can be particularly challenging due to competing nationalistic priorities [45] and resource constraints [27,35]. Beyond the health sector, our findings emphasize the importance of the broader policy environment in determining the health outcomes of the population. Ultimately, the population’s lack of legal status in Malaysia inhibits their access to formal employment and education, thus becoming a determinant to the multiple barriers that they face in accessing healthcare services. Overall, this highlights the need for a multi-sectoral approach in effectively addressing the social determinants of refugees’ health. Many studies are in line with this proposition, stating that solutions to improve the health of refugee populations lie not only within the health sector but also with other sectors [46,47,48] such as the immigration, labour and finance sector.

Our findings identified four main strategies in progressing towards a better response to refugee health issues in Malaysia: to adopt a comprehensive health advocacy strategy grounded in the right to healthcare for all; to tackle the social determinants of health; to seek diversified funding to ensure refugee health needs are met; and to improve coordination between the various actors across sectors. These strategies align closely with global goals encapsulated in the 2030 Sustainable Development Agenda which envisions to leave no one behind, particularly the most excluded and marginalized groups of society including refugees and asylum-seekers [49]. In line with the development of a Global Compact for Safe, Orderly and Regular Migration (GCM) by the United Nations [50], the World Health Assembly had also endorsed a resolution in May 2017 urging member states to strengthen bilateral and international cooperation to provide necessary health-related assistance to countries hosting large populations of refugees [51]. Additionally, in a ASEAN Consensus on the Protection and Promotion of the Rights of Migrant Workers, *“those who become undocumented through no fault of their own”* are also included [52]. Through the consensus, receiving states renewed their commitment to the provision of adequate medical and healthcare to migrant populations in accordance with local laws [52].

Malaysia is not a state party to the 1951 Convention Relating to the Status of Refugees and its’ 1967 Protocol [5,6]. Nonetheless, the proposed strategies from our findings provide opportunities for Malaysia to actualize its various domestic and foreign policies. Firstly, prioritizing the less fortunate is deeply ingrained in the MoH’s organisational objective. In line with this, the MoH is committed to realizing the peoples’ *“full potential in health”* through an equitable, affordable and high-quality health system [53]. These goals can be translated through the implementation of an inclusive and equitable health policy that extends coverage to the most vulnerable groups among the population in Malaysia including refugees and asylum-seekers. Secondly, Malaysia envisions to evolve into a society with strong moral and ethical values for social advancement through many of its past and existing initiatives [54,55]. It is therefore timely for Malaysia to devise a policy that promotes social inclusion for all. Thirdly, Malaysia has committed to various regional and international policy instruments including the ASEAN Consensus on the Protection and Promotion of the Rights of Migrant Workers [52]; Responsibility to Protect (RtoP) [56]; the Convention of the Rights of Children (CRC) [57]; and the Convention on the Elimination of All Forms of Discrimination Against Women (CEDAW) [58]. These commitments can serve as a stepping stone to extend protection to vulnerable populations such as refugees and asylum-seekers who may comprise as subgroups that fall under the scope of these policy instruments. Lastly, the humanitarian crisis that forced more than half a million Rohingya refugees out of Myanmar to Bangladesh [59] has also seen efforts from the Malaysian government through its’ foreign policy. The Malaysian government had shown its commitment through the delivery of humanitarian aid, the dispatch of volunteers as well as the assignment of an attaché at Malaysian High Commission in Dhaka to manage relief programs, in addition to building a field hospital at the Cox’s Bazaar’s camp [60,61]. These goodwill policies will also see positive impact on refugee health in Malaysia if implemented domestically.

Finally, in advocating for the health rights of refugees and asylum-seekers, many participants also emphasized that UHC could be a potential strategy to ensure equitable healthcare among the refugee and asylum-seeker population on the basis that access to healthcare is a fundamental human right. Therefore, a future migrant friendly policy could consider the inclusion of such vulnerable populations in the overall provision of UHC at the country level.

### Strengths and Limitations

This is the first study to examine the health systems responses and challenges in addressing the health needs of refugees and asylum-seekers in Malaysia. This study’s findings provide novel insight on the challenges that health actors encounter in addressing refugee health issues, the health systems responses and suggested ways forward that may inform policy and interventions for refugee health within the Malaysian context and beyond. A strength of the study is in the data collection process which involved capturing the viewpoints of a wide range of health actors. The use of semi-structured interviews also enabled the exploration of issues that emerged beyond the scope of the interview guide.

However, this study has several limitations. The participants in this study were predominantly from civil society. Only two participants from public healthcare facilities were interviewed and none from the private sector. Furthermore, the sample of participants is comprised of individuals who were interested in the topic area and outspoken in their views on the issue. This may have limited the breadth of perspectives as participants were mostly sympathetic to the refugee cause. Nonetheless, most participants maintained objectivity in their views by providing a holistic presentation of the issues discussed. A second limitation of the study was the potential bias in interpreting the results since two of the researchers (FLHC, TST) hold an ‘insider’ position in this study as they share similar professional experiences with the participants, having worked on refugee health and social programs in Malaysia, and a third author (JY) is currently being employed by UNHCR. To minimize bias, self-reflexivity was emphasized throughout the study to challenge the influence of pre-conceived ideas, assumptions and subjectivities on how findings were interpreted. In addition, the involvement of the fourth researcher (HLQ) brought great value to the study in this respect as her experience was more relevant to migrant health issues in Europe. This researcher played a key role in ensuring that all topics were covered throughout the data collection phase and that all researchers analysed the study findings objectively. Finally, changes in the political landscape following the study period were not accounted for in the present study’s findings.

## 5. Conclusions

In Malaysia, refugees and asylum-seekers often face multiple barriers in accessing healthcare, leading to poor health outcomes among the population. Our study identified a wide range of actors involved in the response to refugee health locally, of which a greater alignment of interests, collaboration and sharing of responsibility is needed. From a health systems and policy perspective, financial constraints remain a key challenge in providing healthcare to the refugee and asylum-seeker population. Due to high healthcare costs, financial difficulties are also a key barrier faced by refugees and asylum-seekers in accessing healthcare. Cultural and language discordance are also key challenges faced by healthcare workers in the provision of healthcare, thus accentuating the need for greater cultural competence and language support. Despite these challenges, healthcare professionals are strongly influenced by the professional norms of providing treatment regardless of one’s legal status and ability to afford. Nonetheless, improved access to medication is needed for those with chronic illnesses in order to effectively address the comprehensive health needs of the refugee population. On the whole, strategies forward can include adopting a comprehensive health advocacy strategy, tackling the social determinants of health, seeking diversified funding for refugee health issues, and improving cross-sectoral collaborative efforts between actors to promote inclusive policies in strengthening the overall response towards refugee health in Malaysia.

## Figures and Tables

**Table 1:** Sample characteristics.

**Organization Type**	***n***
UN organizations	5
Public healthcare facilities	2
International civil society organizations	6
Local civil society organizations	4
Academia	3
Total	20
**Professional Role in relation to Refugee Work**	***n***
Program manager	7
Program executive	2
Policy and programmatic work	3
Healthcare professional	5
Academician	3
Total	20
**Background**	***n***
Clinician	11
Allied health (e.g., pharmacy, psychology, community health)	4
Non-health (e.g., law, economics, operations)	5
Total	20

**Table 2:** Key challenges faced by actors in responding to refugee health issues in Malaysia.

Sector	Key Challenges as Reported by Participants	Selected Quotes
State government	Challenged by budget constraints, resulting in the need to prioritize healthcare for citizens over healthcare for foreigners including refugees and asylum-seekers. Responses to the health needs of the refugee and asylum-seeker population are partly dependent on existing immigration laws. As described, immigration laws may contradict with the professional duties of healthcare workers in treating asylum-seeker patients without documents.	*“The government is also facing difficulties in trying to manage with the restricted budget they have […] I think what they did is probably something that would need to be done for them in managing and ensuring that healthcare is prioritized for Malaysian citizens.” (I09)* *“In theory, public health providers should provide treatment regardless of legal status; but they also have to follow the mandate from the ministry and immigration laws of the country. This places them in a difficult position when dealing with undocumented asylum-seekers.” (I03)*
Civil society	Limited human and financial resources to address the comprehensive health needs of refugees and asylum-seekers. Capacities are further undermined by policy amendments involving increased foreigner fees, as more refugees and asylum-seekers turn to NGO clinics for treatment and medication. Challenges in carrying out public advocacy work due to resource constraints.	*“[NGO services] are very limited and it’s not sustainable because people in the NGO clinic, they change and the manpower usually is very limited.” (I01)* *“I think what’s lacking is people who are a bit more vocal […] of course, we can’t expect our health NGOs to be very local, given the fact that they’d be worried about jeopardizing their own operations as well […] and the limitation of the human resources and time that they have […] that’s limiting a little bit of what they can do, outside of just delivering health services.” (I09)*
UNHCR	UNHCR Malaysia’s budget does not commensurate with the actual health needs of the refugee population. Further budget cuts have occurred in the recent year due to the overall increase in refugee needs worldwide. The increase in medical fees for foreigners at public health facilities has impacted UNHCR’s capacity in delivering financial aid to refugees requiring secondary and tertiary care. The numbers requesting for such assistance has also increased. Cross-sector collaborations may be challenged due to the lack of legal framework for refugees.	*“Yeah, I think the budget [of UNHCR] does not commensurate with the growth in the population and the […] amount of assistance that’s required. Yes, there is the insurance, but it’s still a challenge.” (I09)* *“I think one of the really big issues is the budget was cut yearly by the United Nations. The reason they say […] there are more refugees in Europe, so the money is all going to Europe, but they didn’t realize, that even though Malaysian refugees is not that critical, but they still need financial help, because they are not legally working in Malaysia, so I don’t think the cutting budget should continue.” (I05)* *“[…] the cost of supporting cases, of course, have more than doubled […] the number of requests for assistance has, of course, increased, because what they used to be able to afford is no longer affordable, so they’re asking UNHCR to top up or pay the whole sum for even simple things like delivery which they used to be able to afford.” (I09)*
Refugee communities	CBOs lack legitimacy in their operations and in providing services given that refugees are not accorded legal status in Malaysia. Some refugee groups may not have CBOs or community leaders whom they can go to for assistance. CBOs may lack the capacity or may not be fully empowered to take care of their community members.	*“Some MCH (mother-and-child health) clinics are like that, if they don’t have a document, you can’t register [the patient], so I don’t know what to do, so I say, ’Go and shop around and look for other MCHs that will accept you for the community card.’” (I06)* *“[For] health promotion, I mentioned that we can go through community leaders, but then community leaders also have limited reaching area. They only know the people they know, but then there are many people that are not under the community leader radar. There are some communities that don’t even have a leader.” (I07)*
Private sector	Profit generating model of financing leads to high healthcare costs at private health facilities, in which refugees and asylum-seekers are not able to afford. Private companies offering healthcare insurance operate based on risk pooling and may struggle to sustain such initiatives due to low enrollment rates.	*“[…] the refugee group or the so-called foreign group may have less affordability to access the private setting.” (I07)* *“It’s a good emergency sort of insurance policy, but the uptake is not that good […] the coverage is not that high because, some of them, first of all, they may not be aware.” (I01)*
Academia	Difficulties in securing funding for refugee-related research. Existing research funding are prioritized for health issues concerning citizens. Limited access to information systems and databases containing data on health of the migrant and refugee population.	*“To do research, you must get money […] [Funders] will never give you money for [refugee] work. They won’t. That’s what stops the lecturers from getting or working with UNHCR […] Oh, you’ll never get the funding for it.” (I04)*

**Table 3:** Description of findings according to the six building blocks of the WHO Health Systems Framework.

Building Block	Description of Findings
2.1 Service delivery	The ability of the health system to deliver effective, safe and quality health interventions to the refugee and asylum-seeker population.
2.2 Health workforce	The capacity of the health workforce to work in ways that are responsive, fair and efficient to achieve the best health outcomes for the refugee and asylum-seeker population.
2.3 Information and research	The availability and reliability of health information and data on health determinants, health systems performance and health status in regard to the refugee and asylum-seeker population.
2.4 Medical products and technologies	The access to essential medicines and medical products including vaccines and technologies among the refugee and asylum-seeker population.
2.5 Healthcare financing	The financing mechanisms for health and the adequacy of funds that ensure refugees and asylum-seekers have access to the needed services.
2.6 Leadership and governance	Issues relating to the existing policies and governance mechanisms concerning the health of refugees and asylum-seekers.

Adapted from the WHO’s Framework for Action Report—Everybody’s Business: Strengthening Health Systems to Improve Health Outcomes (WHO, 2007) [24].

## Declarations

Availability of data and materials. The datasets generated during and/or analyzed during the current study are not publicly available due to the sensitivity of the data collected and that data may contain information that could compromise the research participants’ privacy and consent.

## Abbreviations

CBOs—Community based organizations
CRC—Convention of the Rights of Children
CSR—Corporate Social Responsibility
CEDAW—Convention on the Elimination of All Forms of Discrimination Against Women
GCM—Global Compact for Safe, Orderly and Regular Migration
HAART—highly active antiretroviral therapy
MoH—Ministry of Health
NGOs—Non-governmental organizations
NUS-IRB—National University of Singapore Institutional Review Board
RtoP—Responsibility to Protect
TN50—National Transformation 2050 / Transformasi Nasional 2050
UHC—Universal Health Coverage
UNHCR—United Nations High Commissioner for Refugees
WHO—World Health Organization
